# Interaction between known risk factors for head and neck cancer and socioeconomic status: the Carolina Head and Neck Cancer Study

**DOI:** 10.1007/s10552-018-1062-8

**Published:** 2018-08-01

**Authors:** Gaelen Stanford-Moore, Patrick T. Bradshaw, Mark C. Weissler, Jose P. Zevallos, Paul Brennan, Devasena Anantharaman, Behnoush Abedi-Ardekani, Andrew F. Olshan

**Affiliations:** 1 Department of Epidemiology, Cambridge University, Cambridge, UK; 2 Department of Otolaryngology/Head and Neck Surgery, University of California, San Francisco, San Francisco, CA USA; 3 School of Public Health, University of California, Berkeley, Berkeley, CA USA; 4 Department of Otolaryngology/Head and Neck Surgery, University of North Carolina, Chapel Hill, Chapel Hill, NC USA; 5 Department of Otolaryngology, Washington University in St. Louis, St. Louis, MO USA; 6 International Agency for Research on Cancer, Lyon, France; 7 Department of Epidemiology, University of North Carolina-Chapel Hill, Chapel Hill, NC USA

**Keywords:** Case–control studies, Epidemiology, Head and neck cancer, Risk factors, Socioeconomic status, Tobacco, Alcohol

## Abstract

Prior studies of squamous cell carcinoma of the head and neck (SCCHN) have explored the effect of socioeconomic status (SES) as an independent risk factor; however, none have investigated the interaction of known risk factors with SES. We examined this using the North Carolina Head and Neck Cancer Epidemiology Study, a population-based case–control study. Incident cases of SCCHN from North Carolina between 2002 and 2006 (*n* = 1,153) were identified and age, sex, and race-matched controls (*n* = 1,267) were selected from driver license records. SES measures included household income, educational attainment, and health insurance. Logistic regression was used to estimate adjusted odds ratios (OR) and 95% confidence intervals (CI). Current smoking was more strongly associated with SCCHN among those households making < $20,000/year [OR 5.11 (3.61–6.61)] compared to household incomes > $50,000/year [OR 2.47 (1.69–3.25); *p* interaction < 0.001]. Current drinking was more strongly associated with SCCHN in household incomes < $20,000 [OR 2.91 (2.05–3.78)] compared to > $50,000/year [1.28 (0.97–1.58); *p* interaction < 0.001]. Current drinkers with less than high school education or income < $20,000 had nearly threefold odds of never-drinkers in the same SES category [OR 2.91 (2.05–3.78); 2.09 (1.39–2.78), respectively]. Our results suggest that the relationship of smoking and alcohol use may be stronger among those of lower SES.

**Electronic supplementary material:**

The online version of this article (10.1007/s10552-018-1062-8) contains supplementary material, which is available to authorized users.

## Introduction

Tobacco and alcohol use have been shown to be consistent risk factors for head and neck cancer [1–4]. However, as the prevalence of smoking decreases in the United States [5], new risk factors have emerged including human papillomavirus [6], poor oral health [7, 8], and genetic factors [9]. Similarly, low socioeconomic status (SES) has been strongly associated with development of oral cancer in men [10]. A 2015 study using pooled international data from over 23,000 cases examined the effects of income and education on head and neck cancer occurrence. They found that fewer years of education and lower income were associated with an increase in disease development. This risk was attenuated when adjusting for alcohol and tobacco use [11]. Here, as with other studies [12–14], socioeconomic status was examined as an independent risk factor, or adjusted as a confounder. However, it is unclear whether the association of risk factors, such as tobacco and alcohol, is modified by SES. A comprehensive description of the interaction between risk factors and SES may offer new insights into the nature of the interaction and clarify other unrelated effects of SES.

It is possible that individuals with lower SES may be more susceptible to the effects of tobacco, alcohol, and other risk factors. To our knowledge, no study to date has explored the joint effect of socioeconomic status and other known risk factors on squamous cell carcinoma of the head and neck (SCCHN). We examined the potential for interaction between various known risk factors for SCCHN and SES in a large population-based study in a racially diverse population.

## Materials and methods

This study uses data previously collected by the Carolina Head and Neck Cancer Epidemiology Study (CHANCE) [15]. Briefly, CHANCE is a population-based case–control study of patients with newly diagnosed first primary invasive SCCHN between 1 January 2002 and 28 February 2006 in North Carolina, United States.

Cases were 20–80 years of age at the time of diagnosis, residents of a 46-county region in North Carolina, and had never been previously diagnosed with head and neck cancer. Controls were identified through the North Carolina Department of Motor Vehicle driver license records and were frequency matched by age, race, and sex. Contact and cooperation rates were 98 and 82% for cases, and 80 and 61% for controls, respectively. Demographic, lifestyle, oral health, dietary, and other risk factor information was collected using a structured questionnaire during an in-home visit. The study was approved by the Institutional Review Board (IRB) of the University of North Carolina at Chapel Hill, and all participating institutions.

The CHANCE questionnaire was completed by 1,389 eligible cases and 1,396 eligible controls. We excluded 21 cases of lip cancer (1.5% of all cases), 28 cases and 18 controls who specified ‘other race’ (46 or 1.7%), and 68 participants (51 cases and 17 controls) who used a proxy during interview. An additional 136, or 10.6%, of eligible cases and 94, or 6.7%, of eligible controls were excluded for missing covariate information. The final study population in our complete case analysis included 1,153 cases and 1,267 controls.

For the assessment of HPV tumor status, patients who had lip and hypopharynx cancers, those for whom the hospital would not release tumor blocks, and those who had completed only proxy interviews were excluded from p16 tumor immunohistochemistry. All patients with oropharyngeal cancers (*N* = 248) and a random sample of patients with non-oropharyngeal cancers (*N* = 244) (because the relevance of HPV in non-oropharyngeal cancer has not been established [16]) were selected for the evaluation of p16-positivity [17].

### Exposure and covariate definitions

Primary exposures of interest included tobacco use, alcohol intake, oral health status, and measures of SES. Cases were asked about exposures the year prior to diagnosis while controls were asked about current use. Tobacco use was defined as (1) never- [ref], ex-, or current smokers; (2) number of cigarettes per day (never-smoker [ref], 0–19, 20–39, 40+); (3) number of years smoked (4) pack-years smoking (never- [ref], 1–10, 11–19, 20–39, 40–49, 50+) (4) never- [ref]/ever-use of smokeless tobacco. Alcohol use was defined as (1) never- [ref], ex-, or current drinker and (2) number of years consuming beer, wine, or liquor (3) quintiles of cumulative lifetime alcohol consumption [grams (g) of ethanol from beer, liquor, and wine; never-drinker [ref], 1–11,232 g, 11,232–204,496 g, 204,496–927,946 g, and ≥ 927,496 g]. In the United States, a standard alcoholic drink is defined as containing 14 g of alcohol [18], therefore the highest quintile of cumulative alcohol consumption is equivalent to more than 25 drinks per week for 50 years. Self-reported oral health variables, selected based on a previous CHANCE study [15], included (1) history of self-reported tooth mobility, or “teeth loose in their socket due to disease” (yes [ref]/no) and (2) one or more routine (non-emergency) dental visits during the decade prior to SCCHN diagnosis (yes [ref]/no). SES factors included (1) household income (> $50,000 per year [ref], $20,000–$50,000 per year, and < $20,000 per year); (2) education (some college or more [ref], high school graduation or 12 years of education but no further, 11 years or fewer of education), and (3) insurance type [none [ref], private, Medicare/Medicaid, other (including Veterans’ Affairs (VA)/military healthcare, TRICARE/CHAMPUS/CHAMPVA, and “other” indicated on the questionnaire)]. Other factors, which served as potential confounders, included environmental tobacco smoke and family history of cancer. Environmental tobacco was defined as never [ref]/ever being exposed to tobacco smoke in the workplace or home. Family history of cancer was defined as having any first-degree relative with family history of any cancer (yes [ref]/no).

### Statistical analysis

We calculated odds ratios (OR) and 95% confidence intervals (CI) for each exposure and SCCHN risk using unconditional logistic regression. We evaluated multiplicative interaction of tobacco use, alcohol use, and oral health status by SES with an exposure-SES product term. Likelihood ratio tests (LRT) were conducted to compare the models with the multiplicative term to the same model without it. Given low statistical power for interaction analysis, an a priori alpha of 0.10 was used as the significance threshold. All analyses were conducted in Stata v14.2. The primary analyses included the modification of tobacco and alcohol use (never, ex-, current), and oral health status associations by SES, but we also conducted additional analyses on the interaction between cigarette duration of use, smokeless tobacco use, quantity of alcohol use, and oral health risk factors and SES.

Minimally adjusted models included only the joint primary exposures (tobacco or alcohol use or oral health and SES) and the matching factors [age (continuous), race (white, African American), sex (male, female)]. Potential confounders were identified using a directed acyclic graph approach [19]. The fully adjusted model included the matching factors [age (continuous), race (white, African American), sex (male, female)] plus additional covariates: oral health parameters, smokeless tobacco, family history of cancer, and SES factors.

Given the known association between HPV infection and SCCHN, particularly oropharyngeal cancer, initial analyses were repeated, stratifying by HPV positivity. p16 status had only been tested in patients who had oropharyngeal cancers (*n* = 248) and a random sample of patients who had non-oropharyngeal cancers (*n* = 244). p16 was chosen as the marker for HPV based on a priori analyses in a previous CHANCE study that determined using p16, rather than both HPV DNA PCR and p16 expression, did not change point estimates [8]. HPV-positive tumors were compared to controls and, separately, HPV-negative tumors were compared to controls, assessing of odds of SCCHN within each stratum of SES. The effect of SES was investigated after stratification by tumor site. Cases within each tumor site were compared to all controls.

A sensitivity analysis was undertaken to compare the results of the primary analysis with reclassification of insurance category. The primary main effect and interaction models with the insurance category were compared to the same models with a re-categorization of insurance. Medicaid and Medicare insurance types were subdivided, to determine the specific effects and interactions related to the two insurance types separately.

## Results

Among cases, 76.3% were male and 74.4% identified as white. Among controls, 69.4% were male and 80.8% identified as white (Table 1). At the time of the interview, cases were slightly younger than controls [mean (median) age of cases: 58.9 (59) years, controls: 61.5 (63)]. The distribution of the primary cancer site for cases (of 1,153) was 163, (14%) oral, 317 (27%) oropharyngeal (OPC), 52 (5%) hypopharyngeal, 416 (36%) laryngeal, and 205 (18%) not otherwise specified. Of all cases who had tumors tested for p16 as a marker of HPV infection, 44% (192 of 434) were p16-positive. A total of 144 (of 248, 58%) of OPC cases were p16 positive. Of non-oropharyngeal cases, 19 (20%) of laryngeal cases, 14 (22%) of oral cavity cases, and 15 (26%) of NOS cases were p16 positive.

**Table 1:** Participant demographics

	Cases (*n* = 1,289)		Controls (*n* = 1,361)	
	*n*	%	*n*	%
**Sex**				
M	984	76.34	945	69.43
F	305	23.66	416	30.86
**Age (years)**				
20–49	253	19.63	156	11.46
50–54	200	15.52	160	11.76
55–59	216	16.76	206	15.14
60–64	217	16.83	205	15.06
65–69	174	13.50	241	17.71
70–74	141	10.94	227	16.68
75–80	88	6.83	166	12.20
Mean (median)	58.9	(59)	61.5	(63)
**Race**				
White	959	74.40	1,100	80.79
African American	330	25.60	261	19.18
**Income**				
> $50,000	354	27.46	584	42.91
$20,000–$50,000	434	33.67	476	34.97
< $20,000	443	34.37	251	18.44
Missing	58	4.50	50	3.67
**Education**				
Less than high school	431	33.44	211	15.50
High school graduate	367	28.47	329	24.17
Some college and above	491	38.09	821	60.32
**Insurance**				
Private	470	36.60	555	40.81
Medicaid/Medicare	432	33.64	430	31.62
None	162	12.62	75	5.51
Other	220	17.13	300	22.06
Missing	5	0.39	1	0.07
**Primary tumor site**				
Oral cavity	183	14.20		
Oropharynx	349	27.08		
Hypopharynx	59	4.58		
Larynx	461	36.54		
NOS	237	18.39		
**Cumulative alcohol consumption (ethanol grams)**				
Median	574,430		701,182	
Mean (SD)	1,377,719	(2,375,966)	389,263	(926,246)
Missing	82 (6.36%)		45 (3.30%)	
**P16 status** ^a^				
P-16 positive	192	44.24		
P-16 negative	242	55.76		

^a^P16 status was only tested in cases who had oropharyngeal cancers (*n* = 248) and a random sample of cases who had non-oropharyngeal cancers (*n* = 244)

### Main effects of tobacco, alcohol, and oral health variables

Table 2 presents the description of known risk factors for SCCHN development, fully adjusted for the matching factors of age, race, and sex, plus pack-years smoking history, lifetime alcohol consumption, smokeless tobacco use, family history of cancer, and oral health parameters (except the exposure of interest). Current smokers were four times more likely to develop SCCHN compared to never-smokers [OR 4.16 (3.21–5.39)]. Longer duration of cigarette smoking and greater number of cigarettes smoked daily were strongly associated with increased odds of SCCHN [OR smoking 50 or more years: 5.83 (3.97–8.57); OR 50 or more cigarettes daily: 3.80 (2.28–6.32)] compared to never-smokers. Lifetime total alcohol consumption was positively associated with odds of SCCHN, with almost four times greater odds of SCCHN in individuals with the highest lifetime consumption of alcohol compared to never-drinkers [OR 3.69 (2.59–5.24)]. Oral health factors, including self-reported history of a loose permanent tooth and lack of routine dental visit within the decade preceding SCCHN diagnosis were both associated with increased odds of SCCHN, respectively [OR 1.48 (1.20–1.83) and OR 1.84 (1.45–2.31)].

**Table 2:** Relationship of known risk factors and odds of SCCHN in CHANCE, 2002–2004

	Cases (*n* = 1,289)		Controls (*n* = 1,361)		Minimally adjusted OR^a^ (95% CI)	Fully adjusted OR^b^ (95% CI)
	*n*	%	*n*	%		
**Smoking status**						
Never-smoker	170	13.19	521	38.28	Ref	Ref
Ex-smoker	382	29.64	572	42.03	2.09 (1.67–2.63)	1.51 (1.17–1.95)
Current smoker	737	57.18	268	19.69	7.84 (6.20–9.91)	4.16 (3.21–5.39)
Years smoked						
Never-smoker	170	13.19	521	38.28	Ref	Ref
1–19	116	9.00	290	21.31	1.12 (0.83–1.51)	1.02 (0.75–1.39)
20–39	491	38.09	328	24.10	4.21 (3.31–5.36)	2.64 (2.03)
40–49	330	25.60	142	10.43	8.76 (6.57–11.69)	4.55 (3.32–6.22)
50 or more	182	14.12	80	5.88	11.08 (7.34–15.85)	5.83 (3.97–8.57)
**Number of cigarettes per day**						
Never-smoker	170	13.19	521	38.28	Ref	Ref
1–19	225	17.46	332	24.39	1.94 (1.49–2.52)	1.49 (1.13–1.98)
20–39	580	45.00	380	27.92	4.79 (3.80–6.04)	2.85 (2.20–3.67)
40–49	228	17.69	97	7.13	7.70 (6.64–10.50)	3.82 (2.71–5.37)
50 or more	86	6.67	31	2.28	8.53 (5.30–13.73)	3.80 (2.28–6.32)
**Pack-years smoking history**						
Never-smoker	170	13.19	521	38.28	Ref	Ref
1–10	102	7.91	235	17.27	1.22 (0.89–1.67)	1.08 (0.78–1.49)
11–19	87	6.75	133	9.77	1.91 (1.34–2.70)	1.50 (1.04–2.17)
20–39	260	20.17	216	15.87	3.60 (2.75–4.72)	2.29 (1.71–3.07)
40–49	159	12.34	78	5.73	6.51 (4.62–9.19)	3.83 (2.65–5.54)
50 or more	511	39.64	178	13.08	10.26 (7.87–13.39)	5.38 (4.02–7.20)
**Alcohol use status**						
Never-drinker	121	9.42	289	21.28	Ref	Ref
Ex-drinker	440	43.24	318	23.42	3.08 (2.33–4.07)	1.64 (1.20–2.25)
Current drinker	724	56.43	751	55.30	1.92 (1.49–2.48)	1.28 (0.95–1.71)
Missing	4	0.31	3	0.22		
**Total alcohol consumption (ml of ethanol)**						
Never-drinker	121	9.39	289	21.23	Ref	Ref
Up to 11,232	57	4.42	160	11.76	0.78 (0.53–1.15)	0.69 (0.46–1.05)
11,232–204,469	231	17.92	404	29.68	1.40 (1.07–1.89)	1.12 (0.82–1.53)
204,469–927,946	315	24.44	320	23.51	2.62 (1.96–3.49)	1.69 (1.22–2.32)
927,946, and greater	565	43.83	188	13.81	8.84 (6.45–12.13)	3.69 (2.59–5.24)
Missing	82	6.36	45	3.30		
**Smokeless tobacco use (chew and/or snuff)**						
Never	1,063	82.47	1,182	86.85	Ref	Ref
Ever use of chew of snuff	226	17.53	179	13.15	1.32 (1.05–1.66)	1.03 (0.79–1.33)
**Environmental tobacco exposure (home and work)**						
None	142	11.03	222	16.32	Ref	Ref
Cigarettes, cigars or pipe	1,145	88.97	1,138	83.68	1.76 (1.38–2.25)	0.97 (0.73–1.28)
Missing	2	0.15	1	0.07		
**History of any Cancer in first-degree relative**						
No	522	40.50	573	42.10	Ref	Ref
Yes	767	59.50	788	57.90	1.30 (1.10–1.54)	1.33 (1.10–1.61)
**Oral health factors**						
Self-reported tooth mobility						
No	801	62.14	1,048	77.00	Ref	Ref
Yes	481	37.32	311	22.85	2.17 (1.80–2.60)	1.48 (1.20–1.83)
Missing	10		4			
Routine dental visits						
Yes	808	62.68	1,145	84.13	Ref	Ref
No	473	36.70	216	15.87	3.17 (2.59–3.88)	1.84 (1.45–2.31)
Missing	11	0.85	0	0		

^a^Adjusted for matching factors (sex, race, age)

^b^Adjusted for matching factors (sex, race, age) plus pack-years smoking history, total lifetime alcohol consumption, smokeless tobacco use, family history of cancer, and oral health parameters (except the exposure of interest). No simultaneous adjustment was undertaken within type of risk factor, i.e., current smoking status was not adjusted for pack-year smoking history and current alcohol use was not adjusted for lifetime total alcohol consumption. All estimates are based on complete case analysis

### Main effects of SES variables

Marked differences in income were noted between cases and controls (Table 3). Years of education were inversely associated with odds of SCCHN, with participants who attained less than a high school education having nearly four times the odds of SCCHN, compared to those who completed some college or more (Table 3, OR 3.97 (3.18–4.96)). Adjustment for tobacco use, alcohol use, and oral health factors, which could be potential mediators, further attenuated the contribution of SES to odds of SCCHN. The odds were higher with minimal adjustment (only matching factors, OR 3.97 (3.18–4.96)) versus adjustment for matching factors, plus family history of cancer, and other SES variables [OR 1.81 (1.35–2.42)].

**Table 3:** Adjusted Odds Ratios for SES and SCCHN characteristics in CHANCE, 2002–2004 (*n* = 2,420: 1,153 cases, 1,267 controls)

	Minimally adjusted^a^		Intermediate adjustment^b^		Fully adjusted^c^	
	OR	95% CI	OR	95% CI	OR	95% CI
**Income**						
> $50,000	Ref	–	Ref	–	Ref	–
$20,000–$50,000	1.73	1.42–2.11	1.38	1.11–1.70	1.13	0.90–1.43
< $20,000	3.63	2.88–4.58	2.23	1.68–2.95	1.56	1.14–2.14
**Education**						
Some college and above	Ref	–	Ref	–	Ref	–
High school graduate	1.89	1.55–2.31	1.62	1.31–2.00	1.26	1.00–1.60
Less than high school	3.97	3.18–4.96	2.91	2.28–3.72	1.81	1.35–2.42
**Insurance**						
Private	Ref	–	Ref	–	Ref	–
None	2.29	1.68–3.14	1.21	0.86–1.72	0.81	0.55–1.19
Medicare/Medicaid	1.77	1.40–2.24	1.00	0.77–1.31	0.85	0.63–1.14
Other	1.32	1.02–1.71	1.11	0.85–1.46	1.02	0.75–1.37

^a^Adjusted for matching factors: age, sex, race

^b^Adjusted for matching factors: age, sex, race plus family history of cancer, and SES (education, income, or insurance type), excluding the SES parameter of interest

^c^Adjusted for matching factors plus total alcohol consumption, duration of cigarette smoking, oral health parameters, smokeless tobacco, family history of cancer, SES factors: income, education, insurance type (except for the SES parameter of interest)

Stratified by tumor site, there was a general trend of increasing odds of SCCHN with lower income and fewer years of education. For example, SCC of the oral cavity had the strongest association with low income and less education [OR 2.24 (1.21–4.14); OR 1.43 (0.86–2.39) for income < $20,000 and less than high school education, respectively] (Supplemental Table 1).

### Interaction between tobacco use and SES

Table 4 shows the interaction results between ever-smoking cigarettes and SES variables. Compared to never-smokers with an annual income greater than $50,000, individuals who were current smokers and had an income less than $20,000 had more than five times the odds of SCCHN [OR 5.11 (3.61–6.61)], while current smokers who had incomes > $50,000 had more than double the odds of SCCHN [OR 2.47(1.69–3.25) *p* interaction < 0.001]. We observed a statistically significant interaction between smoking status and education level (*p* interaction 0.009), and for smoking status and insurance type (*p* interaction 0.011). Current smokers with less than a high school education had seven times the odds of never-smokers with some college education [OR 7.38 (5.03–9.73)]. Current smokers who attended some college or more were at nearly 2.5 times the odds of SCCHN than never-smokers with some college education [OR 2.49 (1.85–3.12)].

**Table 4:** Interaction between ever-smoking cigarettes and SES variables (*n* = 2,420: 1,153 cases, 1,267 controls)

	Fully adjusted^a^			
	Never-smoker *n* = 651 (156 cases, 495 controls)	Ex-smoker *N* = 856 (338 cases, 518 controls)	Current smoker *N* = 913 (659 cases, 254 controls)	*p* Interaction
	OR (95% CI)	OR (95% CI)	OR (95% CI)	
**Income**				< 0.001
> $50,000	1 (Ref)	1.17 (0.84–1.50)	2.47 (1.69–3.25)	
$20,000–$50,000	0.83 (0.52–1.14)	1.28 (0.94–1.62)	3.77 (2.77–4.78)	
< $20,000	0.84 (0.43–1.24)	2.11 (1.39–2.85)	5.11 (3.61–6.61)	
**Education**				0.009
Some college and above	1 (Ref)	1.30 (0.98–1.62)	2.49 (1.85–3.12)	
High school graduate	0.93 (0.55–1.30)	1.53 (1.10–2.00)	4.45 (3.14–5.78)	
Less than high school	0.91 (0.38–1.44)	2.09 (1.36–2.81)	7.38 (5.03–9.73)	
**Insurance type**				0.011
Private	1 (Ref)	1.30 (0.93–1.67)	2.68 (1.92–3.45)	
Medicaid/Medicare	0.47 (0.26–0.67)	1.09 (0.76–1.42)	3.26 (2.27–4.26)	
None	1.03 (0.19–1.88)	1.40 (0.37–2.43)	2.05 (1.25–2.85)	
Other	0.89 (0.46–1.32)	1.03 (0.67–1.40)	4.36 (2.43–6.29)	

^a^Adjusted for matching factors plus total alcohol consumption, oral health parameters, smokeless tobacco, family history of cancer, SES factors: income, education, insurance type (other than the of parameter interest)

Current smokers with Medicaid/Medicare had the greatest odds of SCCHN compared to never-smokers with private health insurance [OR 3.26 (2.27–4.26)]. In contrast, current smokers with private insurance had two times the odds of SCCHN compared to never-smokers with private insurance [OR 2.68 (1.92–3.45)].

Although no evidence of interaction was observed between duration of cigarette smoking and SES (*p* interaction: 0.24, 0.16, and 0.14 for income, education, and insurance, respectively) a suggestive pattern exists at the highest categories of smoking duration and income, similar to the income contrast for current smokers. Compared to never-smokers making more than $50,000 per year, the OR for smoking cigarettes for 40 or more years and having an income less than $20,000 was 5.52 (3.74–7.29) (Supplemental Table 2), which was of greater magnitude than for individuals with 40 or more years of smoking history with an income greater than $50,000 [OR 3.40 (2.12–4.69)]. Similarly, smoking 40 or more years and having less than high school education was associated with nearly seven times the odds of SCCHN [OR 6.91 (4.61–9.21)]; for those in the same smoking category with some college education or more, the odds ratio was 3.92 (95% CI 2.67–5.17) (Supplemental Table 2). There was no clear pattern of interaction between smokeless tobacco by SES (Supplemental Table 3).

Table 5 presents the interaction between alcohol consumption by SES variables. The models showed evidence of multiplicative interaction between currently drinking alcohol and having lower income or fewer years of education (*p* values for interaction: 0.0693 and 0.0269, respectively). Current drinkers with incomes less than $20,000 were at nearly three times the odds of SCCHN compared to never-drinkers with income less than $20,000 (OR 2.91; 95% CI 2.05–3.78), while current drinkers with incomes greater than $50,000 had a less pronounced OR [1.28 (0.97–1.58)]. Individuals who had the lowest income and who drank the most alcohol, corresponding to approximately 25 drinks per week for 50 years, had nearly six times the odds of SCCHN compared to never-drinkers in the highest income tertile [OR 5.85 (3.74–7.96)] (Supplemental Table 4). This is greater than for those individuals with the same drinking history but an annual household income greater than $50,000 [OR 3.44 (1.98–4.90)]. Individuals with less than high school education and the highest category of alcohol consumption had five times the odds of SCCHN [OR 5.25 (3.27–7.23)], while individuals with some college education and the same drinking history having three times the odds [OR 2.95 (1.92–3.99)]. However, we found no evidence of interaction between the three SES variables and total grams of alcohol consumption (*p* values for interaction: 0.47, 0.54, and 0.60 for interaction with income, education, and insurance, respectively).

**Table 5:** Interaction between ever-drinking alcohol and SES variables (*n* = 2,420: 1,153 cases, 1,267 controls)

	Fully adjusted^a^			
	Never-drinker *N* = 390 (114 cases, 276 controls)	Ex-drinker *N* = 678 (396 cases, 282 controls)	Current drinker *N* = 1,352 (643 cases, 709 controls)	*p* Interaction
	OR (95% CI)	OR (95% CI)	OR (95% CI)	
**Income**				0.069
> $50,000	1 (Ref)	2.16 (1.37–2.95)	1.28 (0.97–1.58)	
$20,000–$50,000	1.25 (0.75–1.75)	1.71 (1.24–2.17)	1.62 (1.24–1.99)	
< $20,000	1.21 (0.66–1.76)	2.52 (1.73–3.32)	2.91 (2.05–3.78)	
**Education**				0.027
Some college and above	1 (Ref)	1.65 (1.17–2.14)	1.04 (0.82–1.26)	
High school graduate	0.82 (0.46–1.18)	1.66 (1.11–2.19)	1.78 (1.33–2.27)	
Less than high school	1.29 (0.66–1.91)	2.19 (1.52–2.87)	2.69 (1.80–3.58)	
**Insurance type**				0.149
Private	1 (Ref)	1.91 (1.28–2.54)	1.56 (1.17–1.95)	
Medicaid/Medicare	0.93 (0.53–1.33)	1.72 (1.21–2.22)	1.37 (0.99–1.75)	
None	1.04 (0.20–2.06)	0.87 (0.40–1.35)	1.97 (1.15–2.80)	
Other	1.24 (0.54–1.94)	2.34 (1.33–3.34)	1.51 (1.04–2.00)	

^a^Adjusted for matching factors plus duration of cigarette smoking, oral health parameters, smokeless tobacco, family history of cancer, environmental tobacco smoke, SES factors: income, education, insurance type (other than the of parameter interest)

The oral health variables, history of loose permanent tooth, and prior routine dental visit, were explored separately (Supplemental Table 5). Neither oral health variable modified risk factor associations (*p* values for interaction of loose tooth: 0.96, 0.14, 0.90 with income, education, and insurance, respectively; *p* value for interaction of routine dental visit: 0.80, 0.66, 0.93 with income, education, and insurance, respectively).

Table 6 presents the odds of SCCHN across levels of income, education, and insurance, stratified by HPV status. HPV-negative individuals with an income less than $20,000 were at nearly two times greater odds of SCCHN development than HPV-negative individuals with incomes greater than $50,000 [OR 1.84 (1.09–3.10)]. HPV-positive individuals in the same income category, less than $20,000, had slightly lower odds [OR 1.35 (0.78–2.35)]. Increasing years of education was associated with decreased risk of SCCHN for both HPV-positive and HPV-negative individuals. However, these models are only based on a subset of the cases who were tested for HPV, therefore are more imprecise and are only suggestive. There were no patterns observed for insurance.

**Table 6:** Risk of SCCHN by SES variables, stratified by HPV status

	Fully adjusted^a^	
	p16 status	
	Positive *N* = 192^b^	Negative *N* = 242^b^
	OR (95% CI)	OR (95% CI)
**Income**		
> $50,000	1(Ref)	1 (Ref)
$20,000–$50,000	1.22 (0.75–1.67)	1.12 (0.72–1.74)
< $20,000	1.35 (0.78–2.35)	1.84 (1.09–3.10)
**Education**		
Some college and above	1 (Ref)	1 (Ref)
High school graduate	1.07 (0.71–1.62)	1.74 (1.15–2.62)
Less than high school	1.48 (0.87–2.51)	2.12 (1.31–3.40)
**Insurance type**		
Private	1 (Ref)	1 (Ref)
Medicaid/Medicare	0.77 (0.46–1.30)	0.92 (0.57–1.51)
None	0.85 (0.45–1.62)	0.72 (0.40–1.31)
Other	0.85 (0.50–1.45)	1.15 (0.68–1.97)

^a^Adjusted for matching factors plus total alcohol consumption, cigarette use duration, family history of cancer, oral health parameters, SES factors: income, education, insurance type (other than the of parameter interest)

^b^Compared to 1,267 controls

Interaction between smoking and drinking status by SES was also explored after additional stratification by HPV status (Supplemental Tables 6 and 7). Among HPV-negative cases, current smokers with an income less than $20,000 were at 10 times the odds of SCCHN when compared to never-smokers with high income [OR 10.76 (6.73–14.79)]. This effect of smoking is higher than in patients with HPV-positive tumors in the same income stratum [OR 1.89 (0.84–2.93)]. However, there was no evidence of interaction (*p* value for interaction: 0.53 and 0.19 for HPV negative and positive, respectively). A similar trend was seen for current smokers with the lowest education level, though there was no statistically significant evidence of interaction (*p* values for interaction: 0.15 and 0.87 in HPV negative and positive, respectively). We did not observe interaction between SES and alcohol use when stratified by HPV status.

In a sensitivity analysis, the original insurance category, containing both Medicare and Medicaid, was subdivided to explore the individual effects. Medicaid was found to be more highly associated with SCCHN development compared to Medicare, although the Medicare results are imprecise. [OR 1.65 (0.91–3.02) and OR 0.74 (0.54–1.01) in Medicaid and Medicare, respectively]. Current smokers with Medicaid had nearly eight times the odds of SCCHN development compared to never-smokers with private health insurance [OR 7.90 (1.09–14.72)], which was a stronger association than observed for those with Medicare [OR 2.68 (1.78–3.57)]. These analyses were limited by the small number of individuals in some cells, but could be explained by differential coverage by the insurance types, such a discrepancy in dental care.

## Discussion

We found evidence of interaction between smoking status, drinking status, and three SES variables (income, education, and insurance). The general pattern reflected stronger associations for factors in the presence of lower SES versus higher SES. As annual household income decreased, there were higher odds of SCCHN in current smokers, drinkers, and with increasing quantity of both cigarettes and alcohol, compared to those with a higher level of SES at the same level of exposure. The odds of SCCHN among heavy smokers and drinkers were highest among those who had the fewest years of education, at the same category of consumption, suggesting a synergistic effect between SES and tobacco or alcohol use. Individuals who had a routine dental visit in the decade prior to SCCHN diagnosis were at decreased odds of SCCHN with increasing years of education. A clear pattern with insurance status was not seen.

We have observed a suggestive pattern of lower SES more strongly associated with risk among cases with HPV-negative tumors than among patients with HPV-positive tumors, although the differences were not statistically significant. There did not appear to be evidence of interaction between smoking and alcohol consumption by SES variables when stratified by HPV status, though sample sizes were very limited. However, there were clear patterns of increased odds of SCCHN in HPV-negative individuals compared to HPV-positive individuals within the same household income or education category at the same levels of tobacco use, when compared to controls. More research with a larger number of cases with HPV data is needed to explore the interrelationship between SES, HPV, and tobacco.

Our results for the association with risk factors agree with reports from the International Head and Neck Cancer Epidemiology Consortium (INHANCE) [11, 20, 21]. Also consistent with our results, prior research has shown that factors related to low SES are associated with increased SCCHN risk [12–14]. For example, a 2015 INHANCE report, which pooled 31 studies from 27 countries and included data from CHANCE, found those with low education had more than twice the odds of SCCHN compared to those with high education [11]. Additionally, a 2008 meta-analysis of socioeconomic inequality and oral cancer risk, which examined 41 studies case–control studies, found twice the odds of oral cancer with low SES, defined by income, occupation, and education [10]. However, no prior studies examined the interaction between SES and known risk factors for SCCHN.

It has been suggested that the association between fewer years of education and/or low income and disease development operate through pathways related to behavioral lifestyle factors or psychosocial factors [22]. Krieger et al. examined multiple theories surrounding the interaction of the individual with the environment, political system, and health and suggested that interactions between all three may lead lower SES groups to be at higher risk for disease [22]. It is possible that this interplay between environment and behavioral factors underlies the increased odds of SCCHN in our study population.

The independent effects of SES could be further explained by residual confounding or other unmeasured risk factor exposures, and misclassification of SES. It is unlikely that there are unmeasured confounding SCCHN risk factors because the etiology of SCCHN has been thoroughly investigated. Potential contributors to the modification of risk factors by socioeconomic status include unmeasured factors such as differing ways cigarette smoke is inhaled, type of cigarette smoked, and measurement errors in reporting of tobacco and alcohol use. Some variation in the effect of SES may be due to measurement error. In our study, each SES variable was classified into three to four categories, possibly resulting in heterogeneity within categories after collapsing information on income, education, or insurance. Additionally, the questionnaire collected income data in categories rather than asking for a fixed amount. Often individuals’ income or health insurance will vary over a lifetime; however for this study, data on the income and insurance type were only collected on the date of interview. We opted to not create an aggregate SES index due to prior literature suggesting the integration of different and complex SES factors could lead to a dilution of data quality [23]. SES is a complex concept and measurement by three variables may not capture all dimensions.

Our study is among the largest individual population-based studies of head and neck cancer conducted in the United States. The population-based design gives us more confidence that our results are generalizable. The questionnaire covered an extensive range of exposures and risk factors, only some of which are discussed in this paper. Additionally, this study is unique in its high proportion of black participants. Other studies of SCCHN have been predominately non-Hispanic whites. In analyzing the effects of SES, it is imperative to include diversity in both race and income. Although the number of African American participants in our study is larger than prior studies, the relatively small sample size of African Americans led to very imprecise estimates in the three-way interaction between race, SES, and known risk factors.

We have opted to not account for multiple comparisons. Given that our analysis is the first to examine this research question, and that we have provided specific a priori hypotheses including interaction, we are providing a broader interpretation of results with a focus on the strength of association and precision of the effect measures [24, 25].

As socioeconomic status becomes more widely recognized as a pertinent factor in SCCHN etiology and prognosis, more measures of socioeconomic status should be collected, such as SES over the life course, and family size and household composition to increase comprehensiveness and precision of socioeconomic status measurement. Furthermore, prior research suggests that SES factors measured should be studied at not only the individual level but also at the contextual and neighborhood level in order to better characterize socioeconomic position [26]. Additionally, future studies should attempt to fully integrate race and ethnicity information, as prior research has demonstrated that the effects of race cannot be fully explained by differences in consumption of tobacco and alcohol between races [27]. In addition, more research is needed on differences in smoking behavior, cigarette type, and access to cessation counseling among different socioeconomic classes.

Given that current smokers and drinkers with lower education and lower income are at significantly higher risk for SCCHN than current smokers and drinkers in higher-income groups, it is important to tailor preventative and interventional measures to these groups. Clinicians should be intimately aware of the patient population they are serving and think of earlier intervention in smoking and drinking for those at higher risk, particularly cessation. Similarly, patient education on SCCHN may be more beneficial if targeted to these high-risk groups. As routine dental visits were found to be protective against SCCHN development and showed increased protection and interaction even within the lowest SES categories, clinicians may consider discussing dental visits with their patients. Ultimately, upstream or distal attempts to decrease poverty, increase educational attainment, and provide adequate health insurance may have increased health benefits beyond that of smoking and drinking cessation.

## Electronic supplementary material

Below is the link to the electronic supplementary material.

Supplementary Tables (DOCX 34 KB)
