# Supplementary material for: Interaction between known risk factors for head and neck cancer and socioeconomic status: the Carolina Head and Neck Cancer Study
Source: Cancer Causes Control. 2018 Aug 1;29(9):863–73. doi: 10.1007/s10552-018-1062-8 (PMC6133180; doi:10.1007/s10552-018-1062-8)
Supplement: Supplementary file 1 — Supplementary Tables (DOCX 34 KB) [file 10552_2018_1062_MOESM1_ESM.docx]

Supplemental Tables for:

**Interaction Between Known Risk Factors for Head and Neck Cancer and Socioeconomic Status: the Carolina Head and Neck Cancer Study**

Gaelen Stanford-Moore MD MPhil*, Cambridge University, Department of Epidemiology and UC San Francisco School of Medicine

Patrick T. Bradshaw PhD, University of California, Berkeley, School of Public Health

Mark C. Weissler, MD, University of North Carolina, Chapel Hill, Department of Otolaryngology/Head and Neck Surgery

Jose P. Zevallos, MD, MPH, Department of Otolaryngology, Washington University

Paul Brennan, PhD, MSc, International Agency for Research on Cancer, Lyon, France

Devasena Anantharaman, PhD, MSc, International Agency for Research on Cancer, Lyon, France

Behnoush Abedi-Ardekani, MD, International Agency for Research on Cancer, Lyon, France

Andrew Olshan PhD, University of North Carolina-Chapel Hill Department of Epidemiology

*corresponding author: Gaelen Stanford-Moore MD MPhil, UC San Francisco School of Medicine, 505 Parnassus Ave, San Francisco, CA 94143, gaelen.stanford-moore@ucsf.edu, +1 (626) 695-1273

ORCID: 0000-0002-2247-8000

**Supplemental Table 1. SES variables stratified by tumor site**

|  | Hypopharynx  N=52^a^ | Larynx  N=416^a^ | Oral Cavity  N=163^a^ | Oropharynx  N=317^a^ | NOS  N=205^a^ |
| --- | --- | --- | --- | --- | --- |
|  | Odds Ratio^b^  (95% CI) | Odds Ratio^b^  (95% CI) | Odds Ratio^b^  (95% CI) | Odds Ratio^b^  (95% CI) | Odds Ratio^b^  (95% CI) |
| Income |  |  |  |  |  |
| >$50,000 | Ref | Ref | Ref | Ref | Ref |
| $20,000-$50,000 | 0.99  (0.39-2.52) | 1.25  (0.88-1.76) | 1.52  (0.92-2.52) | 0.89  (0.63-1.27) | 1.18  (0.78-1.77) |
| <$20,000 | 1.80  (0.64-5.10) | 1.40  (0.91-2.15) | 2.24  (1.21-4.14) | 1.64  (1.04-2.60) | 1.12  (0.66-1.92) |
|  |  |  |  |  |  |
| Education |  |  |  |  |  |
| Some college and above | Ref | Ref | Ref | Ref | Ref |
| High School graduate | 0.95  (0.39-2.30) | 1.37  (0.98-1.92) | 1.24  (0.83-1.85) | 1.61  (1.00-2.58) | 1.22  (0.86-1.73) |
| Less than high school | 1.81  (0.73-4.51) | 2.10  (1.42-3.07) | 1.43  (0.86-2.39) | 2.05  (1.20-3.50) | 1.77  (1.14-2.75) |
|  |  |  |  |  |  |
| Insurance |  |  |  |  |  |
| Private | Ref | Ref | Ref | Ref | Ref |
| None | 1.68  (0.59-4.77) | 0.78  (0.47-1.32) | 1.00  (0.50-1.97) | 0.56  (0.32-0.96) | 1.09  (0.59-2.00) |
| Medicare/Medicaid | 1.58  (0.59-4.22) | 1.11  (0.73-1.69) | 1.07  (0.60-1.92) | 0.53  (0.33-0.82) | 1.26  (0.75-2.11) |
| Other | 0.82  (0.24-2.80) | 1.22  (0.79-1.88) | 1.53  (0.84-2.81) | 0.65  (0.41-1.02) | 1.60  (0.95-2.69) |

^a^Compared to 1,267 controls ^b^Adjusted for matching factors plus total alcohol consumption, cigarette use duration, family history of cancer, oral health parameters, SES factors: income, education, insurance type (other than the of parameter interest)

**Supplemental Table 2. Interaction between duration of cigarette use (years) and SES Variables**

**(n=2,420: 1,153 cases, 1,267 controls)**

|  | Fully Adjusted^a^ | | | |
| --- | --- | --- | --- | --- |
|  | **Never-Smoker**  **N=651** | **1-19 Yrs**  **N= 368** | **20-39 Yrs**  **N= 752** | **40+ Yrs**  **N=649** |
|  | OR  (95% CI) | OR  (95% CI) | OR  (95% CI) | OR  (95% CI) |
| Income |  |  |  |  |
| >$50,000 | 1 (Ref) | 0.87  (0.55-1.20) | 1.86  (1.29-2.42) | 3.40  (2.12-4.69) |
| $20,000-$50,000 | 0.89  (0.55-1.22) | 1.04  (0.61-1.49) | 2.28  (1.68-2.89) | 4.46  (3.05-5.89) |
| <$20,000 | 0.97  (0.50-1.45) | 1.13  (0.35-1.91) | 4.34  (2.82-5.87) | 5.52  (3.74-7.29) |
| Education |  |  |  |  |
| Some college and above | 1 (Ref) | 0.87  (0.58-1.16) | 1.94  (1.44-2.44) | 3.92  (2.67-5.17) |
| High School graduate | 0.99  (0.59-1.40) | 1.51  (0.76-2.25) | 2.82  (1.97-3.68) | 4.36  (2.85-5.88) |
| Less than high school | 1.07  (0.44-1.69) | 1.05  (0.25-1.85) | 4.97  (3.16-6.78) | 6.91  (4.61-9.21) |
| Insurance Type |  |  |  |  |
| Private | 1 (Ref) | 1.0  (0.63-1.35) | 2.38  (1.71-3.05) | 4.89  (2.83-6.96) |
| Medicaid/Medicare | 0.71  (0.39-1.02) | 1.28  (0.55-2.02) | 1.87  (1.23-2.50) | 3.95  (2.74-5.71) |
| None | 0.94  (0.16-1.71) | 0.53  (0.15-1.91) | 2.80  (1.49-4.11) | 1.82  (0.76-2.87) |
| Other | 1.30  (0.67-1.93) | 0.81  (0.28-1.32) | 2.46  (1.48-3.45) | 4.14  (2.45-5.82) |

^a^Adjusted for matching factors plus total alcohol consumption, oral health parameters, smokeless tobacco, family history of cancer, SES factors: income, education, insurance type (other than the of parameter interest)

**Supplemental Table 3. Interaction between ever-use of smokeless tobacco (Chew, snuff) and SES variables**

**(n=2,420: 1,153 cases, 1,267 controls)**

|  | Fully Adjusted^b^ | |
| --- | --- | --- |
|  | **Have you ever used snuff or chew?** | |
|  | **No**  **N= 2,050** | **Yes**  **N=** **370** |
|  | OR  (95% CI) | OR  (95% CI) |
| Income |  |  |
| >$50,000 | 1 (Ref) | 0.84  (0.46-1.21) |
| $20,000-$50,000 | 1.11  (0.87-1.35) | 1.04  (0.63-1.45) |
| <$20,000 | 1.57  (1.19-1.95) | 1.45  (0.79-2.10) |
| Education |  |  |
| Some college and above | 1 (Ref) | 0.96  (0.54-1.39) |
| High School graduate | 1.30  (1.00-1.59) | 1.03  (0.57-1.49) |
| Less than high school | 1.79  (1.32-2.24) | 1.65  (1.00-2.30) |
| Insurance Type |  |  |
| Private | 1 (Ref) | 0.89  (0.51-1.27) |
| Medicaid  /Medicare | 0.94  (0.51-1.27) | 0.50  (0.71-1.18) |
| None | 0.71  (0.45-0.97) | 1.88  (0.01-3.74) |
| Other | 0.95  (0.66-1.24) | 1.40  (0.60-2.20) |

^a^Adjusted for matching factors: age, sex, race ^b^Adjusted for matching factors plus total alcohol consumption, cigarette use duration, family history of cancer, oral health parameters, SES factors: income, education, insurance type (other than the of parameter interest)

**Supplemental Table 4. Interaction between cumulative life-time alcohol consumption and SES variables**

**(n=2,420: 1,153 cases, 1,267 controls)**

|  | Fully Adjusted^a^ | | | |
| --- | --- | --- | --- | --- |
|  | **Never-Drinker**  **N=390** | **1-11,232 grams**  **N= 212** | **11,233-927,946 grams**  **N= 1,220** | **>927,946 grams**  **N=598** |
|  | OR  (95% CI) | OR  (95% CI) | OR  (95% CI) |  |
| Income |  |  |  |  |
| >$50,000 | 1 (Ref) | 0.94  (0.45-1.43) | 1.45  (1.11-1.80) | 3.44  (1.98-4.90) |
| $20,000-$50,000 | 1.27  (0.77-1.76) | 0.81  (0.38-1.24) | 1.59  (1.21-1.96) | 4.28  (2.85-5.70) |
| <$20,000 | 1.28  (0.71-1.90) | 0.72  (0.11-1.33) | 2.83  (1.98-3.69) | 5.85  (3.74-7.96) |
| Education |  |  |  |  |
| Some college and above | 1 (Ref) | 0.56  (0.30-0.83) | 1.23  (0.98-1.49) | 2.95  (1.92-3.99) |
| High School graduate | 0.85  (0.48-1.22) | 1.18  (0.43-1.93) | 1.55  (1.13-1.97) | 4.09  (0.74-2.10) |
| Less than high school | 1.42  (0.74-2.10) | 0.78  (0.12-1.44) | 2.45  (1.68-3.21) | 5.25  (3.27-7.23) |
| Insurance Type |  |  |  |  |
| Private | 1 (Ref) | 0.73  (0.38-1.09) | 1.76  (1.32-2.20) | 4.04  (2.44-5.65) |
| Medicaid  /Medicare | 0.98  (0.56-1.40) | 0.56  (.17-0.95) | 1.45  (1.05-1.86) | 3.68  (2.41-4.94) |
| None | 1.09  (0.03-2.15) | 0.67  (0.06-7.06) | 1.24  (0.70-1.78) | 3.80  (1.63-5.98) |
| Other | 1.25  (0.55-1.94) | 1.96  (0.46-3.47) | 1.64  (1.11-2.16) | 3.49  (1.85-5.12) |

^a^Adjusted for matching factors plus duration of cigarette smoking, oral health parameters, smokeless tobacco, family history of cancer, environmental tobacco smoke, SES factors: income, education, insurance type (other than the of parameter interest)

**Supplemental Table 5. Interaction between oral health factors and SES variables**

**(n=2,420: 1,153 cases, 1,267 controls)**

|  | Fully Adjusted^a^ | | | |
| --- | --- | --- | --- | --- |
|  | **Did you ever have a permanent tooth that was loose in its socket?** | | **Have you ever received a routine dental exam?** | |
|  | **No**  **N= 1,724** | **Yes**  **N=696** | **Yes**  **N=696** | **No**  **N= 1,724** |
|  | OR  (95% CI) | OR  (95% CI) | OR  (95% CI) | OR  (95% CI) |
| Income |  |  |  |  |
| >$50,000 | 1 (Ref) | 1.40  (0.89-1.91) | 1 (Ref) | 1.24  (0.57-1.92) |
| $20,000-$50,000 | 1.12  (0.87-1.37) | 1.58  (1.10-2.06) | 1.10  (0.86-1.36) | 1.63  (1.12-2.14) |
| <$20,000 | 1.62  (1.17-2.05) | 2.13  (1.44-2.83) | 1.52  (1.10-1.94) | 2.34  (1.63-3.13) |
| Education |  |  |  |  |
| Some college and above | 1 (Ref) | 1.67  (1.15-2.20) | 1 (Ref) | 1.37  (0.77-1.98) |
| High School graduate | 1.32  (1.00-1.64) | 1.81  (1.20-2.41) | 1.20  (0.91-1.49) | 2.03  (1.34-2.72) |
| Less than high school | 2.12  (1.53-2.72) | 2.12  (1.42-2.82) | 1.87  (1.30-2.45) | 2.49  (1.74-3.24) |
| Insurance Type |  |  |  |  |
| Private | 1 (Ref) | 1.48  (0.98-1.99) | 1 (Ref) | 1.33  (0.79-1.87) |
| Medicaid  /Medicare | 0.87  (0.65-1.10) | 1.23  (0.83-1.62) | 0.83  (0.61-1.05) | 1.29  (0.88-1.70) |
| None | 0.85  (0.51-1.19) | 1.12  (0.48-1.75) | 0.77  (0.41-1.13) | 1.25  (0.67-1.82) |
| Other | 1.10  (0.75-1.43) | 1.30  (0.74-1.85) | 1.02  (0.72-1.32) | 1.40  (0.65-2.15) |

^a^Adjusted for matching factors plus total alcohol consumption, cigarette use duration, smokeless tobacco, family history of cancer, SES factors: income, education, insurance type (other than the of parameter interest)

**Supplemental Table 6. Fully adjusted OR for interaction between current cigarette use and SES variables, stratified by HPV status**

|  | P16 Positive^a^ | | | P16 Negative^a^ | | |
| --- | --- | --- | --- | --- | --- | --- |
|  | **Never-Smoker**  **N= 48** | **Ex-Smoker**  **N= 68** | **Current Smoker**  **N= 76** | **Never-Smoker**  **N= 24** | **Ex-Smoker**  **N= 55** | **Current Smoker**  **N= 163** |
|  | OR  (95% CI) | OR  (95% CI) | OR  (95% CI) | OR  (95% CI) | OR  (95% CI) | OR  (95% CI) |
| Income |  |  |  |  |  |  |
| >$50,000 | 1 (Ref) | 0.96  (0.56-1.35) | 0.88  (0.41-1.35) | 1 (Ref) | 1.67  (0.70-2.65) | 6.26  (3.06-9.46) |
| $20,000-$50,000 | 0.74  (0.27-1.20) | 1.00  (0.54-1.45) | 1.71  (0.96-2.45) | 1.38  (0.40-2.36) | 1.52  (0.70-2.34) | 7.80  (4.93-10.67) |
| <$20,000 | 0.58  (-0.39-1.20) | 1.39  (0.43-2.34) | 1.89  (0.84-2.93) | 1.35  (0.22-2.48) | 4.04  (1.97-6.13) | 10.76  (6.73-14.79) |
| Education |  |  |  |  |  |  |
| Some college and above | 1 (Ref) | 1.28  (0.81-1.75) | 1.42  (0.83-2.00) | 1 (Ref) | 1.62  (0.81-2.43) | 4.12  (2.35-5.88) |
| High School graduate | 1.12  (0.33-1.89) | 1.22  (0.56-1.88) | 1.80  (0.85-2.75) | 1.46  (0.41-2.52) | 1.67  (0.66-2.68) | 10.18  (6.36-14.01) |
| Less than high school | 1.70  (0.34-0.83) | 0.75  (0.47-1.03) | 0.84  (0.49-1.18) | 0.73  (0.51-1.62) | 3.23  (1.51-4.97) | 11.28  (6.68-15.89) |
| Insurance Type |  |  |  |  |  |  |
| Private | 1 (Ref) | 1.19  (0.75-1.64) | 1.17  (0.65-1.70) | 1 (Ref) | 2.02  (0.91-3.13) | 8.15  (4.76-11.54) |
| Medicaid/Medicare | 0.44  (0.06-0.83) | 0.73  (0.30-1.16) | 1.40  (0.60-2.22) | 0.85  (0.15-1.54) | 2.01  (0.98-3.05) | 7.00  (4.10-9.88) |
| None | 1.59  (0.49-5.16) | 0.41  (0.05-3.50) | 1.14  (0.37-1.90) | 1.66  (0.31-9.00) | 2.92  (0.77-11.05) | 4.24  (1.85-6.62) |
| Other | 0.67  (0.03-1.31) | 0.80  (0.27-1.24) | 1.64  (0.39-2.89) | 2.11  (0.33-3.89) | 1.65  (0.42-2.87) | 9.35  (3.67-15.03) |

^a^adjusted for matching factors plus total alcohol consumption, family history of cancer, oral health parameters, SES factors: income, education, insurance type (other than the of parameter interest)

**Supplemental Table 7. Fully adjusted OR for interaction between current alcohol use by SES variables, stratified by HPV status**

|  | P16 Positive^a^ | | | P16 Negative^a^ | | |
| --- | --- | --- | --- | --- | --- | --- |
|  | **Never-drinker**  **N= 20** | **Ex-drinker**  **N= 60** | **Current drinker**  **N= 112** | **Never-drinker**  **N= 24** | **Ex-drinker**  **N= 82** | **Current drinker**  **N= 136** |
|  | OR  (95% CI) | OR  (95% CI) | OR  (95% CI) | OR  (95% CI) | OR  (95% CI) | OR  (95% CI) |
| Income |  |  |  |  |  |  |
| >$50,000 | 1 (Ref) | 2.27  (0.97-3.57) | 1.40  (0.97-1.85) | 1 (Ref) | 1.12  (0.35-1.88) | 0.71  (0.37-1.05) |
| $20,000-$50,000 | 0.99  (0.23-1.74) | 2.23  (1.21-3.25) | 1.59  (1.21-3.25) | 0.54  (0.09-0.98) | 1.04  (0.55-1.54) | 1.09  (0.68-1.50) |
| <$20,000 | 1.27  (0.13-2.41) | 2.52  (0.99-4.05) | 2.17  (0.90-3.45) | 0.80  (0.20-1.39) | 1.71  (0.93-2.48) | 1.90  (1.13-2.66) |
| Education |  |  |  |  |  |  |
| Some college and above | 1 (Ref) | 1.69  (0.92-2.45) | 0.93  (0.66-1.19) | 1 (Ref) | 1.59  (0.71-2.46) | 1.05  (0.63-1.48) |
| High School graduate | 0.31  (0.07-0.68) | 1.47  (0.59-2.34) | 1.51  (0.88-2.14) | 1.11  (0.24-1.98) | 1.67  (0.75-2.60) | 2.88  (1.82-3.94) |
| Less than high school | 1.03  (0.01-2.05) | 2.03  (0.82-3.22) | 1.66  (0.58-2.73) | 1.59  (0.31-2.88) | 3.20  (1.74-4.66) | 2.37  (1.26-3.47) |
| Insurance Type |  |  |  |  |  |  |
| Private | 1 (Ref) | 2.99  (1.56-4.41) | 1.98  (1.36-2.60) | 1 (Ref) | 1.22  (0.49-1.76) | 1.10  (0.63-1.58) |
| Medicaid/Medicare | 0.97  (0.10-1.83) | 2.11  (0.91-3.30) | 1.41  (0.61-2.20) | 0.39  (0.07-0.72) | 1.28  (0.68-1.87) | 1.10  (0.60-1.60) |
| None | 2.39  (0.49-11.55) | 1.13  (0.28-4.58) | 2.50  (0.78-4.21) | 1.19  (0.20-7.15) | 0.46  (0.05-0.88) | 1.02  (0.41-1.62) |
| Other | 1.32  (0.35-4.96) | 3.09  (0.91-5.26) | 1.38  (0.54-2.22) | 0.90  (0.24-3.34) | 1.78  (0.48-3.08) | 1.13  (0.50-1.77) |

^a^Adjusted for matching factors plus total alcohol consumption, family history of cancer, oral health parameters, SES factors: income, education, insurance type (other than the of parameter interest
